# Morphological and genetic diversity of *Rhipicephalus sanguineus* sensu lato from the New and Old Worlds

**DOI:** 10.1186/1756-3305-6-213

**Published:** 2013-07-23

**Authors:** Filipe Dantas-Torres, Maria Stefania Latrofa, Giada Annoscia, Alessio Giannelli, Antonio Parisi, Domenico Otranto

**Affiliations:** 1 Department of Immunology, Aggeu Magalhães Research Centre, Oswaldo Cruz Foundation 50670420 Recife, Pernambuco, Brazil; 2 Department of Veterinary Medicine, University of Bari, 70010 Valenzano, Bari, Italy; 3 Experimental Zooprophylactic Institute of Apulia and Basilicata, 70017 Putignano, Bari, Italy

**Keywords:** *Rhipicephalus sanguineus*, Taxonomy, Morphology, Phylogeny

## Abstract

**Background:**

The taxonomic status of the brown dog tick (*Rhipicephalus sanguineus* sensu stricto), which has long been regarded as the most widespread tick worldwide and a vector of many pathogens to dogs and humans, is currently under dispute.

**Methods:**

We conducted a comprehensive morphological and genetic study of 278 representative specimens, which belonged to different species (i.e., *Rhipicephalus bursa*, *R. guilhoni*, *R. microplus*, *R. muhsamae*, *R. pusillus*, *R. sanguineus* sensu lato, and *R. turanicus*) collected from Europe, Asia, Americas, and Oceania. After detailed morphological examination, ticks were molecularly processed for the analysis of partial mitochondrial (16S rDNA, 12S rDNA, and *cox*1) gene sequences.

**Results:**

In addition to *R. sanguineus* s.l. and *R. turanicus*, three different operational taxonomic units (namely, *R.* sp. I, *R*. sp. II, and *R*. sp. III) were found on dogs. These operational taxonomical units were morphologically and genetically different from *R. sanguineus* s.l. and *R. turanicus*. Ticks identified as *R. sanguineus* s.l., which corresponds to the so-called “tropical species” (=northern lineage), were found in all continents and genetically it represents a sister group of *R. guilhoni*. *R. turanicus* was found on a wide range of hosts in Italy and also on dogs in Greece.

**Conclusions:**

The tropical species and the temperate species (=southern lineage) are paraphyletic groups. The occurrence of *R. turanicus* in the Mediterranean region is confirmed. A consensual re-description of *R. sanguineus* s.s. and *R. turanicus* will be necessary to solve the taxonomic problems within the so-called *R. sanguineus* group.

## Background

“*Sanguineus, punctatus, postice lineolis tribus impressus; dorso antico macula nulla thoracica, distincta*”, which stands for “blood red, punctate, posteriorly with three impressed lines; no distinct ‘thoracic’ spot anterodorsally”, is all Pierre Andrè Latreille provided in his original description of *Rhipicephalus sanguineus* (Latreille, 1806) [1]. This tick is accounted as the most widespread ectoparasite of dogs and as a well-recognised vector of numerous pathogens to dogs and humans worldwide [2,3]. Latreille’s description was acceptable for that time, when tick taxonomy was in its infancy. However, from today’s perspective, it is a very poor description.

Originally described as ‘*Ixodes sanguineus*’, this tick was reclassified as belonging to the genus *Rhipicephalus* by Koch [4], being the type species of this genus. Later on, several authors strove to study this species group [5-16], whose taxonomy is still the subject of debate [3,15]. According to Camicas and colleagues [15], the so-called “*R. sanguineus* group” includes 17 species as follows: *Rhipicephalus aurantiacus* Neumann, 1907; *Rhipicephalus bergeoni* Morel and Balis, 1976, *Rhipicephalus boueti* Morel, 1957; *Rhipicephalus camicasi* Morel, Mouchet and Rodhain, 1976; *Rhipicephalus guilhoni* Morel and Vassiliades, 1963; *Rhipicephalus leporis* Pomerantzev, 1946; *Rhipicephalus moucheti* Morel, 1965; *Rhipicephalus pumilio* Schulze, 1935; *Rhipicephalus pusillus* Gil Collado, 1936; *Rhipicephalus ramachandrai* Dhanda, 1966; *Rhipicephalus rossicus* Yakimov and Kol-Yakimova, 1911; *R. sanguineus* sensu stricto (s.s.); *Rhipicephalus schulzei* Olenev, 1929; *Rhipicephalus sulcatus* Neumann, 1908; *Rhipicephalus tetracornus* Kitaoka and Suzuki, 1983; *Rhipicephalus turanicus* Pomerantzev, 1940; and *Rhipicephalus ziemanni* Neumann, 1904. However, there is no consensus [3] and the morphological similarities among ticks belonging to the *R. sanguineus* group make their identification a difficult task, even for experienced taxonomists. Moreover, the absence and/or the difficulties in assessing the type specimens of important species further complicate the taxonomical situation within the *R. sanguineus* group.

Many genetic markers have been employed to elucidate the phylogeny and the evolution of *Rhipicephalus* ticks. In particular, the mitochondrial 16S and 12S (rDNA) ribosomal DNA target regions have been frequently used [17-22] and, to a lesser extent, the cytochrome *c* oxidase subunit 1 (*cox*1) and the internal transcribed spacer 2 (ITS-2) have been used [23-27]. Nonetheless, there is still much discussion on the biosystematic status of tick species belonging to this genus.

Over the last decade, several molecular investigations have attempted to assess the genetic variability of *R. sanguineus* sensu lato (s.l.) and to differentiate closely related taxa within the *R. sanguineus* group from different geographical localities [18-22]. These studies have claimed the existence of two divergent lineages within *R. sanguineus* s.l. According to a study, the so-called “southern lineage” (=temperate species) included ticks from localities of Argentina, Uruguay, Chile and Italy, whereas the northern lineage (=tropical species) included ticks from Brazil, Paraguay, Colombia, South Africa, Mozambique, and from two localities of Northern Argentina [22]. Based on preliminary genetic data, the authors suggested that the northern lineage represents a different species from *R. sanguineus* s.s., whereas the southern lineage probably represents the true *R. sanguineus* s.s. However, it has been recognized that further morphological and genetic studies of ticks belonging to the *R. sanguineus* complex from the Old World are necessary to determine the phylogenetic relationship among the taxa of this species group [22]. Importantly, even if most available morphological descriptions of *R. sanguineus* s.s. are somewhat convergent (e.g., [10,14,16,28]), there is no consensual description for this species, especially considering that the type specimen does not exist. In this context, we conducted a comprehensive morphological and genetic study of brown dog ticks from Europe (France, Greece, Italy, Portugal, Spain), Asia (India, Pakistan, Thailand, Turkmenistan, and Vietnam), Africa (Nigeria, and South Africa), the Americas (Brazil, Colombia, Costa Rica, Guatemala, and Honduras), and Oceania (Australia). Our results reveal that the so-called northern lineage includes ticks from all continents and that the southern lineage is not monophyletic. The existence of distinct operational taxonomic units (OTUs) in the Mediterranean region is documented.

## Methods

### Tick collection and identification

The majority of the ticks included in this study were selected from >5,000 specimens examined during previous investigations carried out from 2008 to 2012 in Brazil, Italy, Portugal, Spain, and Greece [29-32]. The remaining specimens were obtained from colleagues from all over the world (see *Acknowledgments*). While most ticks were collected from dogs, specimens from other hosts (e.g., cats, horses, cattle, goats, and sheep) and from the environment were also included (for details, see Table 1), mainly for comparison purposes.

**Table 1:** ***Rhipicephalus *ticks (*n *= 278) included in this study, with data on hosts and geographical origin**

**Species**^**a**^	**Hosts**	**Geographical origin**
*Rhipicephalus bursa* (17/14)	Cattle, goat, sheep	Italy (Basilicata)
*Rhipicephalus guilhoni* (5/5)	Cattle	Nigeria (Plateau State)
*Rhipicephalus microplus* (1/0)	Cattle	Guatemala
*Rhipicephalus muhsamae* (6/6)	Cattle	Nigeria (Plateau State)
*Rhipicephalus pusillus* (3/1)	Rabbit (*O. cuniculus*)	Italy (Sicily)
*Rhipicephalus sanguineus* s.l. (86/65)	Dog	Australia (New South Wales), Brazil (São Vicente Férrer), Colombia (Cali, Medellin), Costa Rica (San Jose), France (south), Guatemala, Honduras (San Pedro), India (Mumbai), South Africa (Cape Province), Thailand (Bangkok), Vietnam (Ho Chi Minh City)
*Rhipicephalus turanicus* (85/54) ^b^	Cattle, horse, goat, dog, cat, Corsican hare (*L. corsicanus*)	Italy (Basilicata, Puglia, Sicily), Greece (Xanthi), Turkmenistan
*Rhipicephalus* sp. I (29/23)	Dog	Italy (Puglia), Greece (Xanthi)
*Rhipicephalus* sp. II (37/24)	Dog	Spain (La Vera), Portugal, Italy (Sicily, Verona)
*Rhipicephalus* sp. III (7/3)	Dog	India (Mumbai), Pakistan (Punjab)
*Rhipicephalus* sp. IV (2/2)	Cattle	Nigeria (Plateau State)

^a^ Numbers within parentheses are the specimens studied morphologically / genetically.

^b^ Also collected from vegetation.

The species identity of each specimen was determined based on morphology, following the keys and descriptions provided by Walker and colleagues [16]. Original descriptions and re-descriptions [5-14,28,33-36] were also used. Furthermore, ticks identified as *R. sanguineus sensu* Walker and colleagues [16] collected from a dog in South Africa (det. Prof. I. G. Horak) and *R. turanicus sensu* Filippova [14] collected by flagging in Turkmenistan (det. Dr. N. A. Filippova) were used as reference specimens. While ticks identified herein as *R. sanguineus* s.l. were compatible with *R. sanguineus sensu* Walker and colleagues [16], they were referred to as *R. sanguineus* s.l., because of the absence of a type specimen and of a consensual species description. Ticks that could not be morphologically assigned to any known species were defined as *Rhipicephalus* sp. and numbered in order of identification as *R*. sp. I, *R*. sp. II, etc. In other words, species defined as *R*. sp. were different from those described in Ref. [16] and from each other, by one or more morphological characters.

Although some of the examined ticks – i.e., *Rhipicephalus bursa* Canestrini and Fanzago, 1878, *Rhipicephalus microplus* (Canestrini, 1888), *Rhipicephalus muhsamae* Morel and Vassiliades, 1965 – are not included into the *R. sanguineus* group, they were also analysed for comparison purposes.

### Morphological study

Out of >5,000 ticks examined, 278 representative specimens of each species were selected and morphologically studied in further detail (Table 1). Ticks were selected based on conservation and feeding status (i.e., only specimens with no obvious indication of engorgement were measured). In some cases, all ticks of a given species (e.g., *R. pusillus*) or from a specific geographical origin (e.g., India) were used, due to the limited number of specimens available. A total of 2,500 pictures were taken from the 278 selected specimens and 31 characters (see below), examined and/or measured, resulting in a database containing over 8,600 entries. Photos were taken using a stereomicroscope equipped with a digital camera linked to a computer. Images were processed and measurements taken (by FD-T) using Leica Application Suite version 4.1 software (Leica Microsystems). Voucher tick specimens are deposited in the Laboratory of Parasitology and Parasitic Diseases of the University of Bari, Italy.

All ticks were carefully observed under a light stereomicroscope, and the following characters were examined and/or measured: idiosoma (length and width); dorsal *scutum* (length and width, punctuation pattern, and shape of posterior margin in females); *basis capituli* (length and width); angles of *basis capituli* (position and shape); hypostomal dentition (number of rows); female porose areas (shape and distance between the two areas); female genital opening (shape); spiracular plates (shape), dorsal tail of spiracular plates (width); first festoon (width); lateral and postmediam grooves (shape); cervical pits (shape); cervical fields (shape); internal and external cervical grooves (shape and punctuation pattern); marginal lines (shape and punctuation pattern); male adanal plates (length, width at base, and presence/absence of median cusps on them); accessory plates (shape); male caudal process (presence/absence); spur on trochanter I (presence/absence); and body colour (pattern). The ratio between the width dorsal tail of spiracular plates, and the width of the adjacent festoon (ST/F1 ratio) was calculated, as well as the ratio between the length and the width (at base) of male adanal plates. These characters are considered taxonomically relevant for *Rhipicephalus* spp. differentiation (e.g., [14,16,17]). Other characters, such as the perforation pattern of the spiracular plates [14,17] were not considered in this study because they may vary between populations [11] and seasonally [37].

### Genetic study

After a detailed morphological study, 197 representative tick specimens were selected for genetic analysis (for details on the number of ticks for each species, see Table 1). DNA extraction was performed using a commercial kit (DNeasy Blood & Tissue Kit, QiagenGmbH, Hilden, Germany), in accordance with the manufacturer’s instructions. Partial mitochondrial 16S rDNA (~300 bp), 12S rDNA (~400 bp), and *cox*1 (~600 bp) gene sequences were generated and analysed. Primers and PCR conditions have been described elsewhere [18,25,38].

Each reaction consisted of 4 μl of tick genomic DNA and 46 μl of PCR mix containing 2.5 mM MgCl2, 10 mM Tris–HCl (pH 8.3), and 50 mM KCl, 250 μM of each dNTP, 50 pmol of each primer and 1.25 U of Ampli Taq Gold (Applied Biosystems). Approximately 100 ng of genomic DNA (with the exception of the no-template control) were added to each PCR. Amplicons were resolved in ethidium bromide-stained (2%) agarose gels (Gellyphor, Italy) and sized by comparison with markers in the Gene Ruler™ 100 bp DNA Ladder (MBI Fermentas, Lithuania). Gels were photographed using a digital documentation system (Gel Doc 2000, BioRad, UK).

PCR products (amplicons) were purified using Ultrafree-DA columns (Amicon, Millipore) and sequenced directly using Taq DyeDeoxyTerminator Cycle Sequencing Kit (v.2, Applied Biosystems) in an automated sequencer (ABI-PRISM 377, Applied Biosystems). The nucleotide sequences obtained were aligned using BioEdit software Version 7.1.3.0 [39] and manually edited. The *cox*1 nucleotide sequences were conceptually translated into amino acid sequences according to the invertebrate mitochondrial code using the MEGA5 software [40].

### Data analyses

All measurements are in millimetres and expressed as mean ± standard deviation. Data comparisons were made considering the tick species for which more than 10 specimens were measured, while others (i.e., *R. guilhoni*, *R. microplus*, *R. muhsamae*, *R. pusillus*, *R.* sp. III, and *R.* sp. IV) were excluded from data analysis. Data was initially assessed using the Lilliefors test for normality and then compared using ANOVA (with Tukey’s post-hoc tests) and a Kruskal-Wallis test (with Dunn’s post-hoc tests), for normal and non-normal data, respectively. Differences were considered significant when *P*<0.05. Statistical analyses were performed using BioEstat 5.0 [41].

The percentage of nucleotide variation among sequences of a given species was calculated by pairwise comparison (Kimura 2-parameter model) [42] using the MEGA5 software [40]. The pairwise comparison of sequence differences (*D*) among *Rhipicephalus* spp. consensus sequences were calculated using the formula *D* = 1 – (*M*/*L*), where *M* is the number of alignment positions at which the two sequences have a base in common, and *L* is the total number of alignment positions over which the two sequences are compared [43].

In order to investigate the phylogenetic relationships among sequences generated from representative tick specimens, we used the neighbour-joining (NJ) [44] and maximum likelihood (ML) methods and the evolutionary distances were computed using the Tajima-Nei and Tamura-Nei models, respectively [45,46]. In addition, the maximum-parsimony (MP) was also run using the close-neighbour-interchange or the subtree-pruning-regrafting algorithms [47]. Phylogenetic analyses were carried out by MEGA5 software [40] and bootstrap values based on 8,000 replicates.

Partial 12S rDNA and 16S rDNA sequences of *Rhipicephalus* spp. available in GenBank were also included. We only used sequences published in reference studies [19-22] or unpublished sequences for which information on host and geographical origin were available. Sequences of *Ixodes ricinus* (L., 1758) available in GenBank (AF150029; JF928527) were used as outgroup. The nucleotide sequences reported in this article have been deposited in the GenBank database (12S rDNA: KC243786-KC243834, KF145151; 16S rDNA: KC243835-KC243871, KF145150; *cox*1: KC243872-KC243931, KF145152, KF145153).

## Results

### Morphological identification

**Figure 1:**
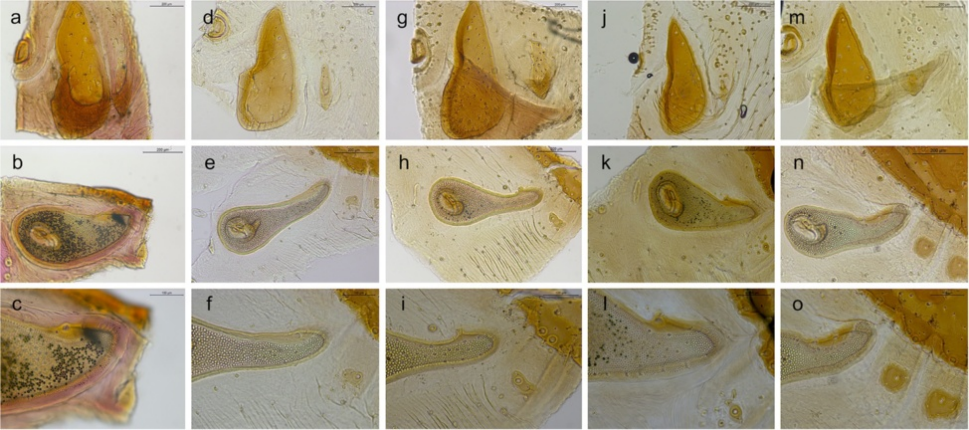
**Distinctive characteristics of male dog-associated *Rhipicephalus *spp.** Adanal plates and accessory shields **(a**, **d**, **g**, **j**, **m)**, spiracular plates **(b**, **e**, **h**, **k**, **n)** and dorsal projection of spiracular plates **(c**, **f**, **i**, **l**, **o)** of *R. turanicus***(a-c)**, *R. sanguineus s.l.***(d-f)**, *R*. sp. I **(g**-**i)**, *R*. sp. II **(j**-**l)** and *R*. sp. III **(m**-**o)**.

**Figure 2:**
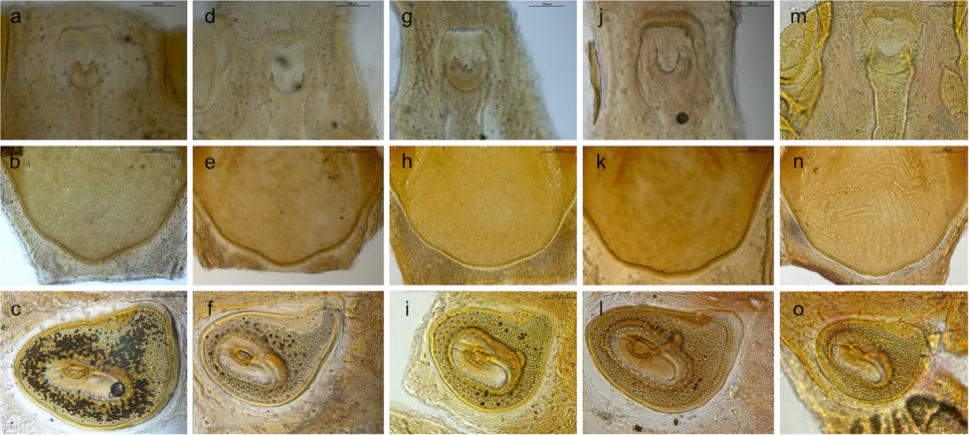
**Distinctive characteristics of female dog-associated *Rhipicephalus *spp.** Genital opening **(a**, **d**, **g**, **j**, **m)**, posterior margin of dorsal *scutum***(b**, **e**, **h**, **k**, **n)**, and spiracular plates **(c**, **f**, **i**, **l**, **o)** of *R. turanicus***(a-c)**, *R. sanguineus s.l.***(d-f)**, *R*. sp. I **(g**-**i)**, *R*. sp. II **(j**-**l)** and *R*. sp. III **(m**-**o)**.

**Figure 3:**
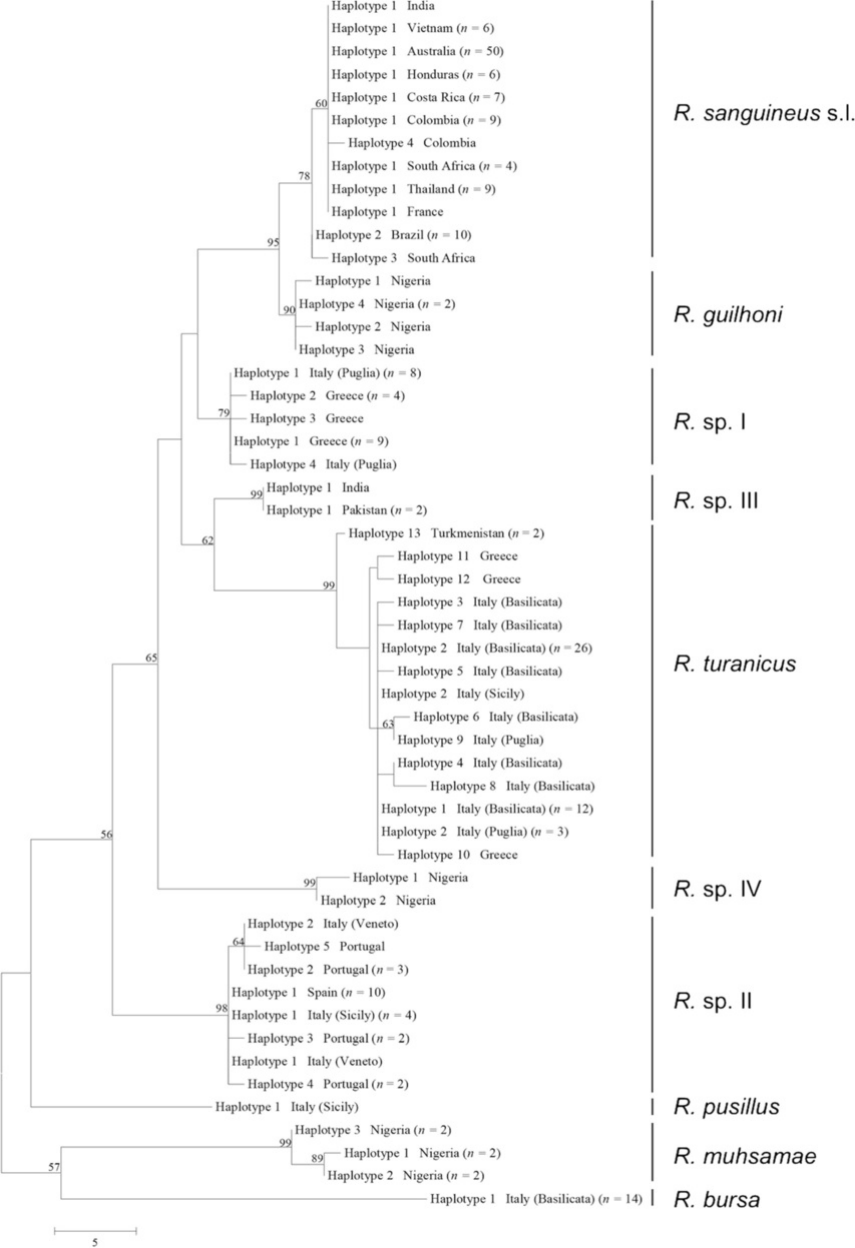
**Phylogeny of *Rhipicephalus *spp. inferred from 16S rDNA.** Maximum-Parsimony tree based on 16S rDNA sequences generated herein. Haplotype, geographical origin and number of specimens from each haplotype are reported. Bootstrap values are based on 8000 replicates and only bootstraps > 50% are indicated.

Seven species were identified: *R. bursa*, *R. guilhoni*, *R. microplus*, *R. muhsamae*, *R. pusillus*, *R. sanguineus* s.l., and *R. turanicus* (Table 1). Remarkably, four OTUs (namely, *R.* sp. I to IV) presenting morphological characters differing from known species and from those above were also found in Italy, Greece, Spain, Portugal, India, Pakistan and Nigeria. These OTUs were closely related to *R. turanicus* and/or *R. sanguineus* s.l., albeit the punctuation pattern on dorsal *scutum*, shape of spiracular plates, adanal plates and accessory shields were distinct. For instance, males of *Rhipicephalus* species found on dogs could be differentiated by comparing the shape of adanal plates, accessory shields, and spiracular plates (Figure 1). Similarly, females of the same species could be distinguished based on genital opening, dorsal scutum, and spiracular plate shapes (Figure 2). Intraspecific morphological variations among ticks identified as *R. sanguineus* s.l. and *R. turanicus* were evident, mainly in terms of colour, size, scutal punctuation pattern, female genital opening shape, spiracular plate shape length, male adanal plate shape and male caudal process (data not shown). However, combining the characteristics mentioned above (Figures 1 and 3), both males and females of *R. sanguineus* s.l. could be separated from *R. turanicus*.

Measurements for key structures of males and female ticks examined in this study are summarized in Table 2. Comparisons of key characteristics of males and females of different species revealed significant differences between some species, as reported in Table 3. However, in most instances, no significant differences were found particularly for close related species (e.g., *R. sanguineus* s.l. and *R.* sp. II).

**Table 2:** **Measurements (mean ± standard deviation) of key structures of *Rhipicephalus *ticks examined in this study**

**Species**	**Sex ( *n *)**	**IL**	**IB**	**SL**	**SB**	**CL**	**CB**	**BCL**	**BCB**	**APL**	**APB**	**AP ratio**	**ST**	**F1**	**ST/F1 ratio**
*R. bursa*	F (6)	6.4±0.2	3.8±0.4	2.5±0.2	2.7±0.2	1.4±0.1	1.6±0.1	0.5±0.1	1.6±0.0	n.a.	n.a.	n.a.	0.1±0.0	0.5±0.1	0.3±0.1
	M (11)	5.7±0.6	3.5±0.4	4.7±0.5	3.1±0.4	1.2±0.1	1.3±0.1	0.5±0.1	1.2±0.1	1.7±0.3	0.9±0.2	1.9±0.2	0.1±0.0	0.3±0.1	0.3±0.1
*R. guilhoni*	F (1)	6.3	4.0	2.3	2.1	1.0	1.3	0.4	1.2	n.a.	na	n.a.	0.2	n.d.	n.d.
	M (4)	4.3±0.1	2.3±0.1	3.5±0.1	2.1±0.1	0.9±0.1	1.0±0.0	0.4±0.0	1.0±0.0	1.2±0.1	0.5±0.0	2.4±0.1	0.2±0.0	0.5±0.7	0.7±0.4
*R. mushamae*	F (2)	6.8±0.1	3.4±0.1	2.6±0.1	2.7±0.0	1.4±0.1	1.4±0.0	0.5±0.0	1.5±0.0	n.a.	n.a.	n.a.	0.2±0.0	0.5±0.0	0.5±0.1
	M (4)	6.2±0.6	3.5±0.3	5.2±0.5	3.3±0.3	1.2±0.1	1.2±0.1	0.5±0.0	1.2±0.1	1.6±0.3	0.7±0.2	2.2±0.2	0.2±0.0	0.4±0.0	0.6±0.1
*R. pusillus*	F (2)	2.3±0.1	1.3±0.1	1.1±0.1	1.0±0.0	0.6±0.0	0.6±0.0	0.2±0.0	0.6±0.0	n.a.	n.a.	n.a.	0.1±0.0	0.2±0.0	0.4±0.1
	M (1)	1.9	1.1	1.5	1.0	0.4	0.5	0.1	0.5	0.4	0.2	2.0	0.0	0.1	0.4
*R. sanguineus* s.l.	F (30)^a^	4.6±0.7	2.6±0.4	1.9±0.2	1.8±0.2	1.0±0.2	1.1±0.2	0.3±0.1	1.0±0.2	n.a.	n.a.	n.a.	0.1±0.0	0.3±0.1	0.5±0.2
	M (55)	4.2±1.0	2.4±0.7	3.4±0.8	2.0±0.5	0.8±0.2	0.9±0.2	0.3±0.1	0.9±0.2	1.0±0.3	0.4±0.1	2.3±0.2	0.1±0.0	0.2±0.0	0.5±0.1
*R. turanicus*	F (33)^a^	4.9±0.7	2.7±0.4	2.1±0.3	2.0±0.3	1.1±0.1	1.2±0.1	0.4±0.1	1.2±0.1	n.a.	n.a.	n.a.	0.2±0.0	0.3±0.1	0.6±0.1
	M (49)	4.7±1.3	2.6±0.8	3.8±1.0	2.3±0.6	0.9±0.3	1.0±0.3	0.4±0.1	1.0±0.3	1.3±0.4	0.5±0.2	2.5±0.2	0.1±0.0	0.2±0.1	0.7±0.1
*R*. sp. I	F (10)	5.5±0.5	3.2±0.4	2.0±0.2	2.0±0.2	1.2±0.1	1.2±0.2	0.4±0.1	1.2±0.1	n.a.	n.a.	n.a.	0.1±0.0	0.3±0.1	0.4±0.1
	M (19)	4.9±0.6	2.9±0.4	3.9±0.5	2.4±0.3	1.0±0.1	1.0±0.1	0.4±0.1	1.0±0.1	1.3±0.3	0.5±0.1	2.4±0.2	0.1±0.0	0.2±0.0	0.4±0.1
*R*. sp. II	F (9)	4.9±1.1	2.7±0.5	2.0±0.4	2.0±0.4	1.1±0.2	1.2±0.3	0.4±0.1	1.1±0.3	n.a.	n.a.	n.a.	0.1±0.0	0.3±0.1	0.5±0.2
	M (28)	5.3±1.0	3.1±0.6	4.3±0.8	2.7±0.5	1.1±0.2	1.2±0.2	0.4±0.1	1.1±0.2	1.4±0.3	0.6±0.1	2.3±0.2	0.1±0.0	0.3±0.1	0.4±0.1
*R*. sp. III	F (3)	4.1±1.9	2.4±1.3	1.6±0.2	1.5±0.4	0.8±0.1	0.9±0.2	0.3±0.1	0.9±0.2	n.a.	n.a.	n.a.	0.1±0.0	0.2±0.0	0.5±0.3
	M (4)	3.3±0.2	2.0±0.2	2.5±0.2	1.6±0.1	0.7±0.0	0.7±0.1	0.2±0.0	0.7±0.1	0.9±0.1	0.4±0.0	2.4±0.1	0.1±0.0	0.1±0.1	1.0±0.2
*R*. sp. IV	F (1)	5.0	2.6	2.3	2.0	1.2	1.2	0.5	1.2	n.a.	n.a.	n.a.	0.3	0.3	0.8
	M (1)	4.3	2.5	3.6	2.0	0.9	1.0	0.4	0.9	1.0	0.5	2.3	0.2	0.2	1.0

^a^An engorged female was excluded.

Abbreviations: *IL:* idiosomal length, *IB:* idiosomal breadth, *SL:* scutal length, *SB:* scutal breadth, *CL:* capitulum length, *CB:* capitulum breadth, *BCL:* basis capituli length, *BCB:* basis capitulum breadth, *APL:* adanal plate length, *APB:* adanal plate breadth, *AP: ratio* adanal plate length/breadth ration, *ST:* dorsal tail of spiracular plate, *F1:* first festoon, *ST/F1:* dorsal tail of spiracular plate/first festoon ratio, *n.a:* not applied, *n.d:* not done.

**Table 3:** **Comparisons **^a^**of key characters for tick species for which enough specimens were available**

**Species**	**I L**	**I B**	**DS C**	**DS B**	**C L**	**C B**	**BC L**	**BC B**	**AP L**	**AP B**	**AP L/B ratio**	**DP B**	**FI B**	**DP/FI ratio**
Rb vs. Rs M	<0.05	**<0.01**	**<0.01**	**<0.01**	<0.05	<0.05	<0.05	<0.05	<0.05	<0.05	<0.01	ns	<0.05	<0.05
Rb vs. Rt M	ns	**<0.01**	**<0.05**	**<0.01**	<0.05	<0.05	ns	<0.05	ns	<0.05	<0.01	ns	<0.05	<0.05
Rb vs. RspI M	ns	**ns**	**ns**	**<0.01**	<0.05	ns	ns	ns	ns	<0.05	<0.01	ns	<0.05	ns
Rb vs. RspII M	ns	**ns**	**ns**	**ns**	ns	ns	ns	ns	ns	ns	<0.01	ns	ns	ns
Rs vs. Rt M	ns	**ns**	**ns**	**<0.01**	ns	<0.05	<0.05	<0.05	<0.05	ns	<0.01	<0.05	ns	<0.05
Rs vs. RspI M	ns	**<0.05**	**ns**	**<0.05**	ns	ns	ns	ns	ns	ns	<0.01	ns	ns	ns
Rs vs. RspII M	<0.05	**<0.01**	**<0.01**	**<0.01**	<0.05	<0.05	<0.05	<0.05	<0.05	<0.05	ns	ns	<0.05	ns
Rt vs. RspI M	ns	**ns**	**ns**	**ns**	ns	ns	ns	ns	ns	ns	<0.01	<0.05	ns	<0.05
Rt vs. RspII M	ns	**<0.05**	**ns**	**<0.05**	ns	ns	ns	ns	ns	ns	<0.01	<0.05	<0.05	<0.05
RspI vs. RspII M	ns	**ns**	**ns**	**ns**	ns	ns	ns	ns	ns	ns	<0.01	ns	ns	ns
Rb vs. Rs F	<0.05	<0.05	<0.05	<0.05	<0.05	<0.05	<0.05	<0.05	−	−	−	ns	<0.01	ns
Rb vs. Rt F	<0.05	<0.05	ns	<0.05	<0.05	<0.05	ns	<0.05	−	−	−	ns	<0.01	<0.05
Rb vs. RspI F	ns	ns	ns	ns	ns	ns	ns	ns	−	−	−	ns	<0.01	ns
Rb vs. RspII F	ns	ns	ns	ns	ns	<0.05	ns	<0.05	−	−	−	ns	<0.01	ns
Rs vs. Rt F	ns	ns	ns	<0.05	ns	<0.05	ns	<0.05	−	−	−	<0.05	ns	<0.05
Rs vs. RspI F	ns	ns	ns	ns	ns	ns	ns	<0.05	−	−	−	ns	ns	ns
Rs vs. RspII F	ns	ns	ns	ns	ns	ns	ns	ns	−	−	−	ns	ns	ns
Rt vs. RspI F	ns	ns	ns	ns	ns	ns	ns	ns	−	−	−	<0.05	ns	<0.05
Rt vs. RspII F	ns	ns	ns	ns	ns	ns	ns	ns	−	−	−	ns	ns	ns
RspI vs. RspII F	ns	ns	ns	ns	ns	ns	ns	ns	−	−	−	ns	ns	ns

^a^Comparisons were made with a Kruskal-Wallis (with Dunn’s post-hoc tests) or ANOVA (with Tukey’s post-hoc; in bold).

Abbreviations: *Rb:Rhipicephalus bursa*, *Rs:Rhipicephalus sanguineus* sensu lato, *Rt:Rhipicephalus turanicus*, *RspI:Rhipicephalus* sp. I, *Rhipicephalus* sp. II, *M:* male, *F:* female, *ns:* not significant, *L:* length, *B:* breadth, *I:* idiosoma, *DS:* dorsal *scutum*, *C:capitulum*, *BC:basis capituli*, *AP:* adanal plates, *DP:* dorsal projection of spiracular plates, *FI:* first festoon.

### Molecular analysis

PCR amplification of each target region from individual DNA samples resulted in amplicons of the expected size, except for *R. microplus* for which no PCR product was obtained. A total of 547 sequences were generated and analysed. The mean level of interspecific pairwise (Pwc) distance (%) among *Rhipicephalus* species was of 10.8% (range, 3.3–18.1%), 9.9% (range, 3.5–15.3%), and 13.3% (range, 9.4–18.7%) for 16S rDNA, 12S rDNA, and *cox*1, respectively (Table 4). Remarkably, a low value of nucleotide difference (i.e., 3.3%, 3.5%, and 9.6%) was registered between *R. guilhoni* and *R. sanguineu*s s.l. for 16S rDNA, 12S rDNA and for *cox*1, respectively (Table 4).

**Table 4:** **Interspecific pairwise (Pwc) distances (%) calculated among consensus sequences for 16S rDNA, 12S rDNA, and *cox*1 haplotypes of each *Rhipicephalus *species examined**

	***R. sanguineus *s.l.**	***R*. sp. I**	***R*. sp. II**	***R*. sp. III**	***R*. sp. IV**	***R. guilhoni***	***R. pusillus***	***R. turanicus***	***R. muhsamae***	***R. bursa***
16S rDNA										
*R. sanguineus* s.l.	-									
*R*. sp. I	5.1	-								
*R*. sp. II	8.7	7	-							
*R*. sp. III	6.2	4	7	-						
*R*. sp. IV	9	7.6	10.5	7.3	-					
*R. guilhoni*	3.3	5.1	9.5	6.2	8.3	-				
*R. pusillus*	9.4	9.4	11.6	10.2	12.6	10.2	-			
*R. turanicus*	11.3	9.5	12.4	8	12	10.6	13.4	-		
*R. muhsamae*	11.6	11.6	12.7	12.7	14.4	11.2	12	15.6	-	
*R. bursa*	15.2	15.2	14.9	14.9	14.1	14.9	15.6	18.1	15.9	-
12S rDNA										
*R. sanguineus* s.l.	-									
*R*. sp. I	10.1	-								
*R*. sp. II	10.5	10.4	-							
*R*. sp. III	7.3	9	7.6	-						
*R*. sp. IV	8.7	9.8	9.3	6.1	-					
*R. guilhoni*	3.5	9.6	9.6	6.7	8.2	-				
*R. pusillus*	9.6	9.6	6.7	6.7	8.1	9.3	-			
*R. turanicus*	10.2	11.3	10.2	8.5	7	9.6	9.9	-		
*R. muhsamae*	11.9	11.3	9.1	8.5	8.4	11.4	7.9	10.7	-	
*R. bursa*	14.4	15.3	13	12.7	13.3	13.6	13.6	13.8	13	-
*cox*1										
*R. sanguineus* s.l.	-									
*R*. sp. I	12.3	-								
*R*. sp. II	15.7	12.5	-							
*R*. sp. III	13.6	9.4	13.2	-						
*R*. sp. IV	12.3	9.4	12.5	10	-					
*R. guilhoni*	9.6	10	14	11.1	11.3	-				
*R. pusillus*	13.8	11.1	14	11.5	9.8	10.9	-			
*R. turanicus*	14.9	11.7	14.2	11.5	12.3	13.6	13.2	-		
*R. muhsamae*	18.7	14.5	16.8	13.8	14.5	15.3	14.2	17	-	
*R. bursa*	16.8	13.8	16.2	14.7	13.8	15.5	13.6	15.5	16.2	-

The overall mean intraspecific variation calculated within each species ranged from 0.3 to 1.2%, depending on genetic target (Table 5). In particular, the mean intraspecific difference for *R. sanguineus* s.l. (from Australia, Brazil, Colombia, Costa Rica, France, Guatemala, Honduras, India, South Africa, Thailand, and Vietnam) ranged from 0.6 to 1.2% (Table 5). Intraspecific differences among sequences were lower than the range of interspecific nucleotide differences (Tables 4 and 5). From one to 22 haplotypes were detected among different species and genes analysed (Table 5).

**Table 5:** **Number of specimens, haplotypes, and intraspecific variation retrieved among 16S rDNA, 12S rDNA, and *cox*1 sequences of each *Rhipicephalus *species examined**

**Species**	***n***	**16S rDNA**		**12S rDNA**		***cox*1**	
		**Haplotypes (no. of specimens)**	**Mean and min-max intraspecific variation (%)**	**Haplotypes (no. of specimens)**	**Mean and min-max intraspecific variation (%)**	**Haplotypes (no. of specimens)**	**Mean and min-max intraspecific variation (%)**
*R. sanguineus* s.l.	65	I (53); II (10); III (1); IV (1)	0.6 (0.4-1.1)	I (50); II (10); III (1); IV (1); V (1)	0.8 (0.3-1.2)	I (25); II (6); III (1); IV (6); V (1); VI (3); VII (1); VIII (3); IX (1)	1.2 (0.2-3.0)
*R*. sp. I	23	I (17); II (4); III (1); IV (1)	0.5 (0.4-0.7)	I (2); II (1); III (1); IV (4); V (7); VI (2); VII (1); VIII (1); IX (1); X (1); XI (2)	0.9 (0.0-2.5)	I (5); II (2); III (1); IV (5)	0.8 (0.2-1.5)
*R*. sp. II	24	I (15); II (4); III (2); IV (2); V (1)	0.7 (0.4-1.1)	I (15); II (1); III (2); IV (2); V (2); VI (1)	0.6 (0.3-1.2)	I (8); II (1); III (2); IV (3); V (1); VI (2); VII (6); VIII (1)	0.6 (0.2-1.1)
*R*. sp. III	3	I (3)	-	I (1); II (2)	0.3	I (1); II (1); III (1)	0.3 (0.4-1.1)
*R*. sp. IV	2	I (1); II (1)	0.7	I (1)	-	I (2)	-
*R. guilhoni*	5	I (1); II (1); III (1); IV (2)	0.5 (0.4-0.9)	I (1); II (2); III (1); IV (1)	0.7 (0.3-1.2)	I (1); II (1); III (1); IV (2)	1.0 (0.2-1.7)
*R. pusillus*	1	I (1)	-	I (1)	-	I (1)	-
*R. turanicus*	54	I (12); II (30); III (1); IV (1); V (1); VI (1); VII (1); VIII (1); IX (1); X (1); XI (1); XII (1); XIII (2)	1.0 (0.4-2.2)	I (36); II (1); III (1); IV (1); V (1); VI (1); VII (1); VIII (2); IX (1); X (1); XI (1); XII (1); XIII (1); XIV (2)	1.0 (0.3-2.2)	I (25); II (1); III (1); IV (1); V (1); VI (1); VII (1); VIII (2); IX (2); X (1); XI (1); XII (1); XIII (1); XIV (1);XV (1); XVI (3); XVII (1); XVIII (2); XIX (1); XX (2); XXI (1); XXII (1)	1.2 (0.2-3.5)
*R. muhsamae*	6	I (2); II (2); III (2)	0.7 (0.4-1.1)	I (1); II (1); III (3); IV (1)	0.5 (0.3-0.9)	I (1); II (1); III (1); IV (1)	1.2 (0.4-1.7)
*R. bursa*	14	I (14)	-	I (13); II (1)	0.3	I (3); II (2); III (1); IV (2); V (1)	0.3 (0.2-0.4)

### Phylogenetic analysis

**Figure 4:**
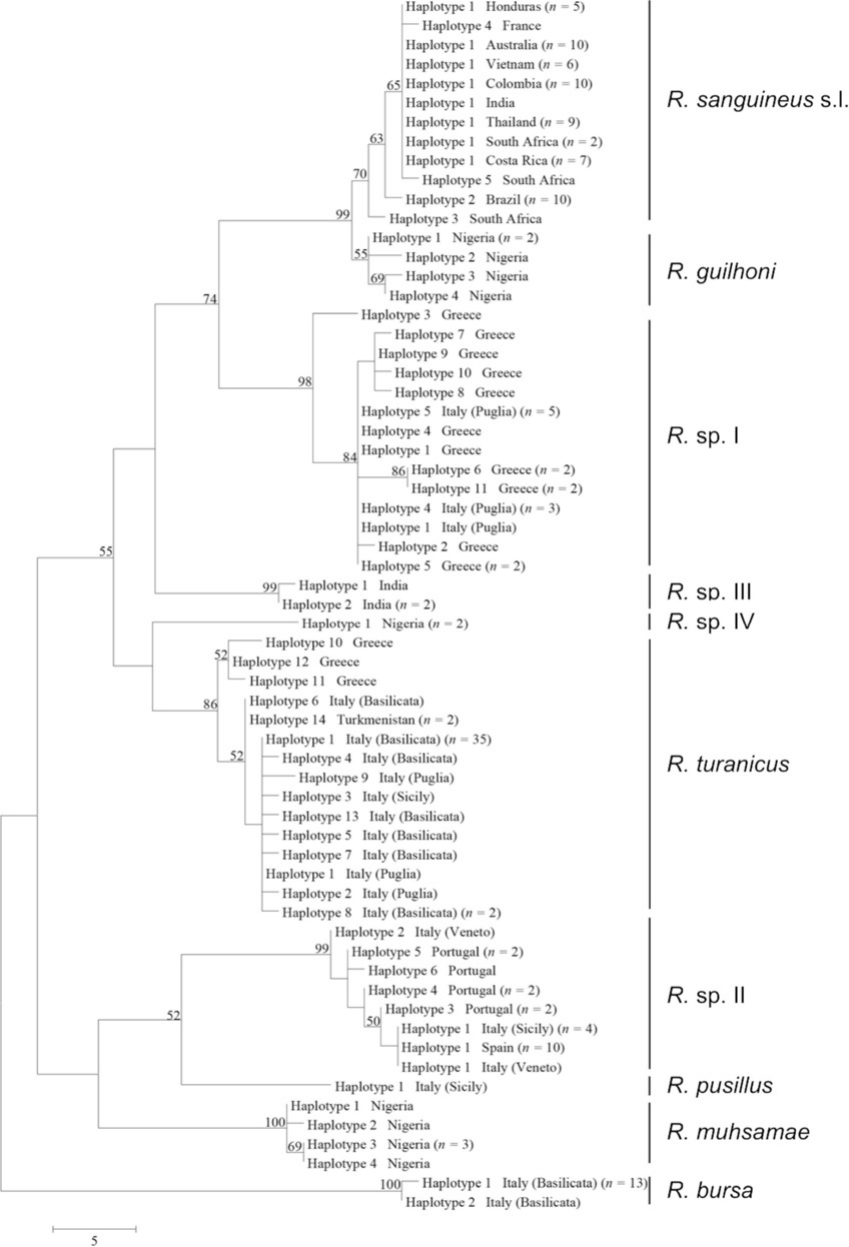
**Phylogeny of *Rhipicephalus *spp. inferred from 12S rDNA.** Maximum-Parsimony tree based on 12S rDNA sequences generated herein. Haplotype, geographical origin and number of specimens from each haplotype are reported. Bootstrap values are based on 8000 replicates and only bootstraps > 50% are indicated.

**Figure 5:**
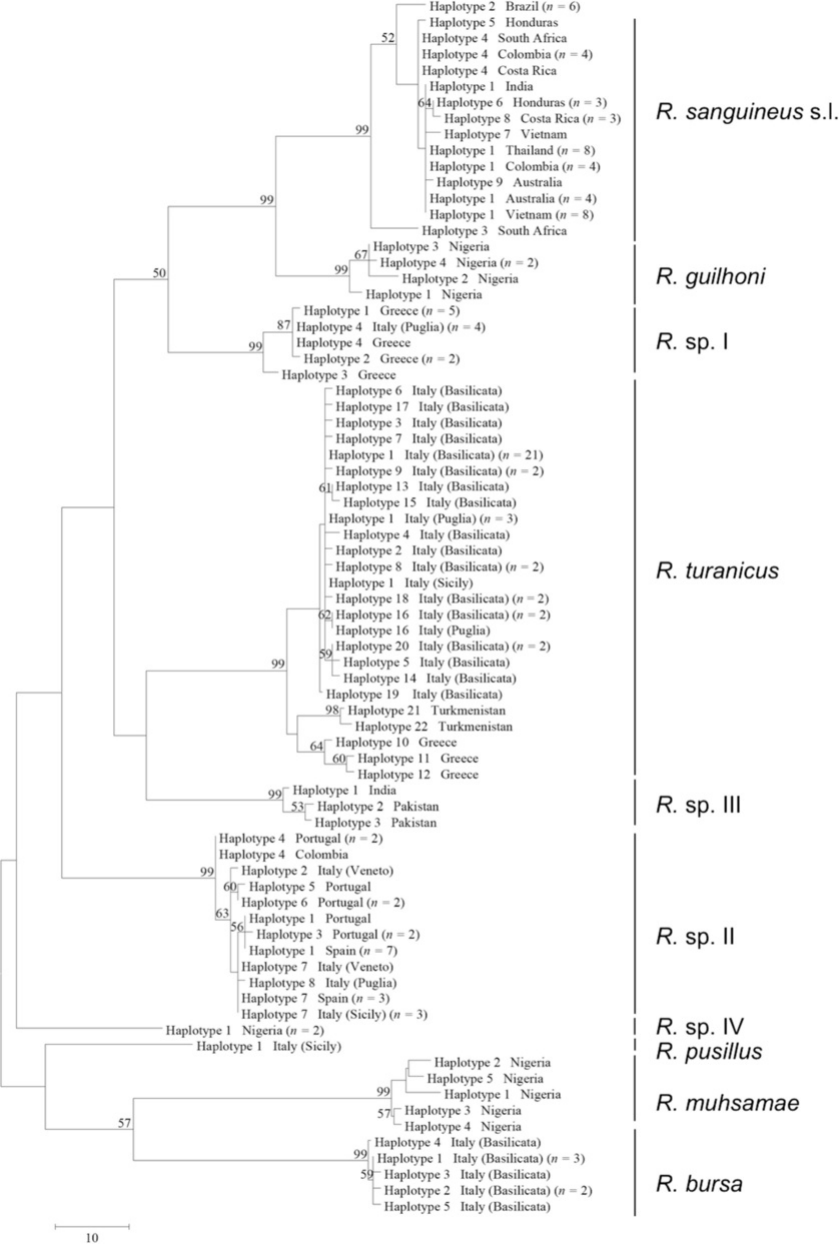
**Phylogeny of *Rhipicephalus *spp. inferred from *cox*1.** Maximum-Parsimony tree based on *cox*1 sequences generated herein. Haplotype, geographical origin and number of specimens from each haplotype are reported. Bootstrap values are based on 8,000 replicates and only bootstraps > 50% are indicated.

Phylogenetic trees (NJ, ML, and MP) of our sequence dataset presented similar topological structures, with high bootstrap values at their main branches (data not shown). Figures 3, 4 and 5 show MP analysis for the three genes examined. In particular, phylogenetic analyses were concordant in clustering *R. sanguineus* s.l. collected from different geographic areas of the New and Old Worlds. Moreover, *R. sanguineus* s.l. was well separated from some of its closest congeners (e.g., *R. turanicus*) and formed a sister group to *R. guilhoni*. Overall, our phylogenetic analyses supported the morphological identification, showing that the four OTUs identified herein belong to different lineages, which are separated from *R. sanguineus* s.l. and *R. turanicus* (Figures 3, 4 and 5).

**Figure 6:**
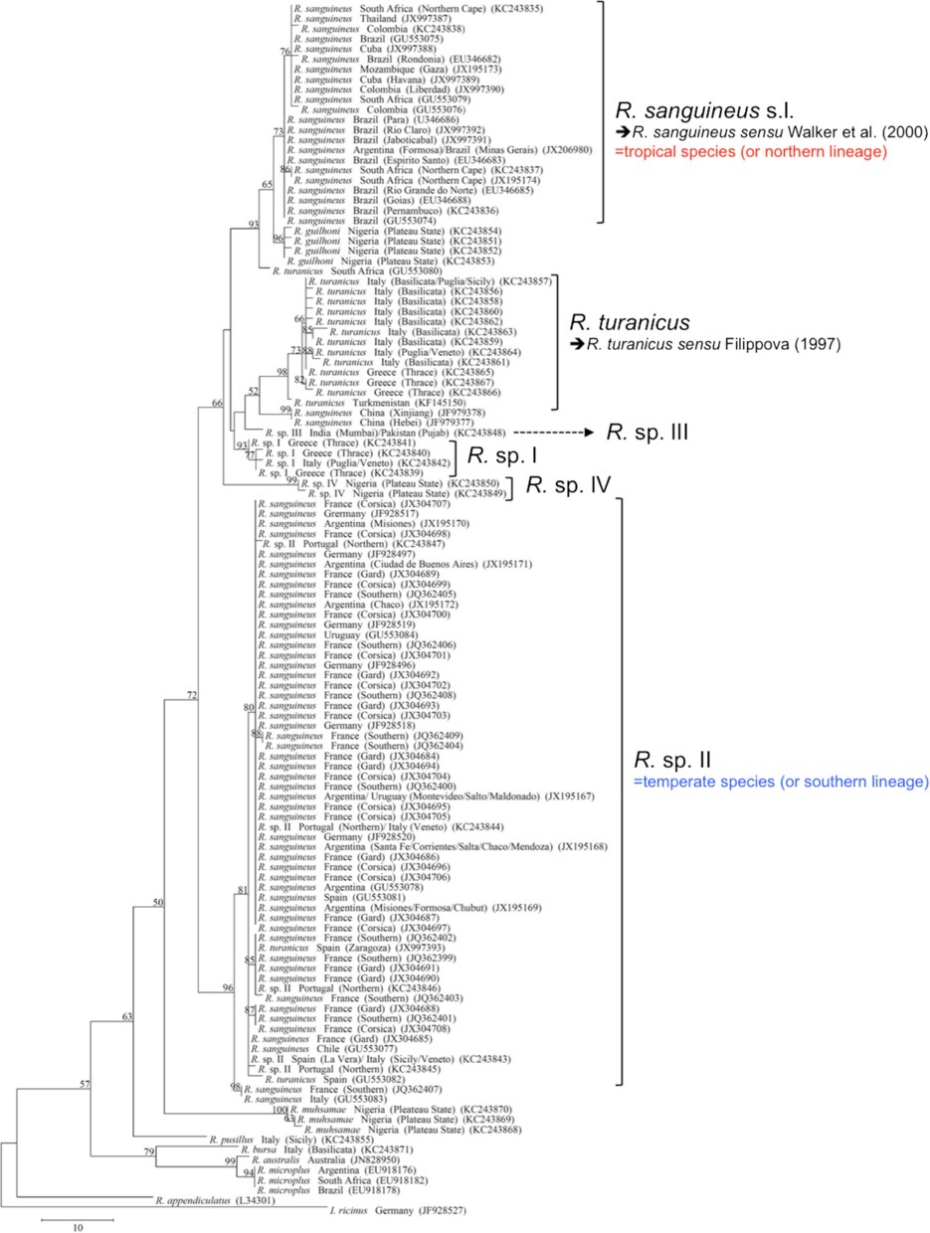
**Phylogeny of *Rhipicephalus *spp. inferred from 16S rDNA (including those available in Genbank).** Maximum-Parsimony tree based on 16S rDNA sequences generated herein along with those from GenBank. Geographical origin and accession numbers are reported. Bootstrap values are based on 8,000 replicates and only bootstraps > 50% are indicated. *Ixodes ricinus* (JF928527) was used as outgroup.

**Figure 7:**
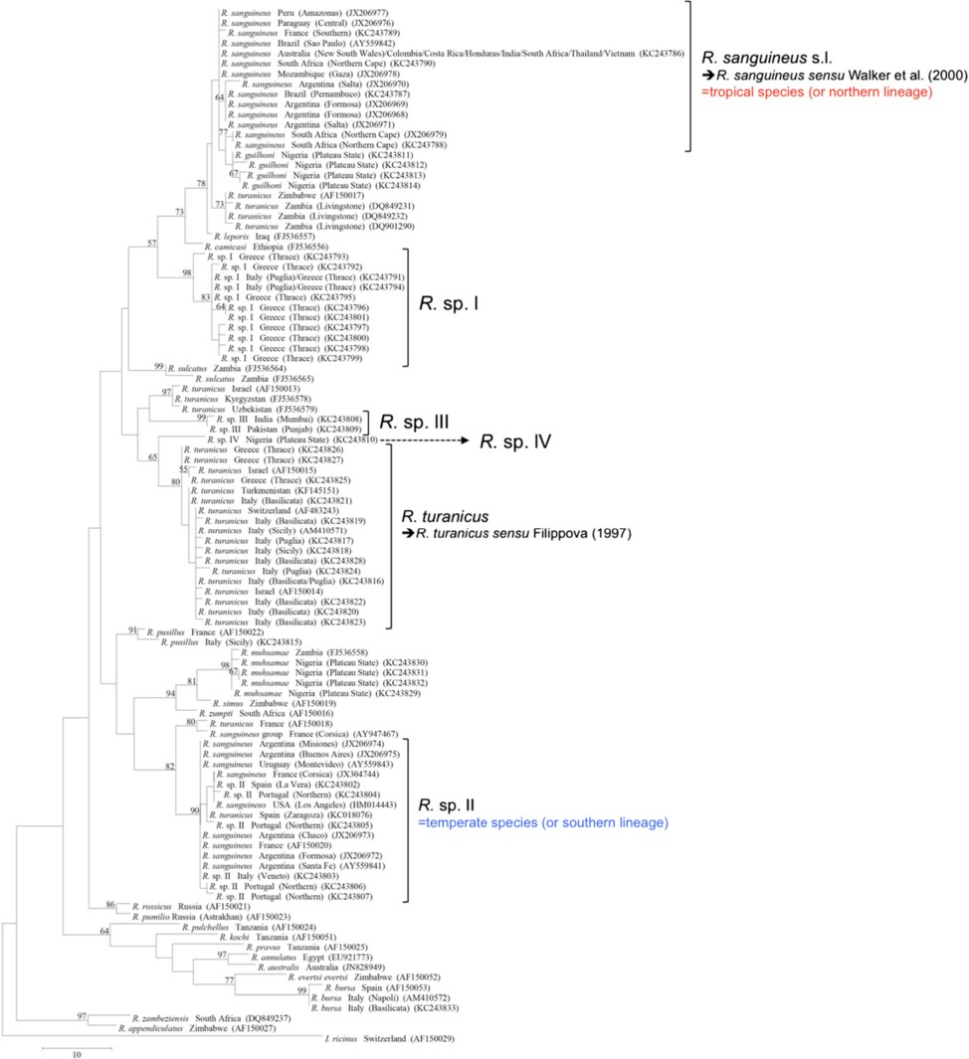
**Phylogeny of *Rhipicephalus *spp. inferred from 12S rDNA (including those available in Genbank).** Maximum-Parsimony tree based on 12S rDNA sequences generated in this study along with those from sequences from GenBank. Geographical origin and accession numbers are reported. Bootstrap values are based on 8,000 replicates and only bootstraps > 50% are indicated. *Ixodes ricinus* (AF150029) was used as outgroup.

The phylogenetic analysis based on edited 12S rDNA and 16S rDNA sequences generated along with those available from recent studies revealed well-defined groups as well (Figures 6 and 7). Ticks identified herein as *R. sanguineus* s.l. fell within the “northern lineage” clade, whereas those identified as *R*. sp. II within a clade that includes the “southern lineage”. The OTUs designated here as *R*. sp. I, *R*. sp. III, and *R*. sp. IV formed independent lineages, even with the inclusion of GenBank sequences (Figures 6 and 7). *R. turanicus* 16S rDNA sequences from Italy and Greece clustered with those from Israel, Switzerland, and Turkmenistan (Figure 6). Remarkably, several 16S rDNA and 12S rDNA sequences labelled as ‘*R. turanicus*’ from GenBank, formed independent lineages or groups (e.g., 16S rDNA: GU553080; 12S rDNA: AF150013, FJ536578, FJ536579 and AF150017, DQ849231, DQ849232, DQ901290) or even fell within the “southern lineage” (i.e., 16S: KC018076; 12S rDNA: JX997393, GU553082) (see Figures 6 and 7). Similarly, some sequences labelled as ‘*R. sanguineus*’ formed independent, well-defined groups (e.g., 16S: JF979377, JF979378).

### Host range and geographical distribution

Ticks identified as *R. sanguineus* s.l. were exclusively found on dogs, whereas *R. turanicus* presented the widest host range, being found on six host species (cattle, goat, horse, dog, cat, and Corsican hares) and also on vegetation. *Rhipicephalus bursa* was found on ruminants (cattle, goat, and sheep), whereas *R. pusillus* was found exclusively on European rabbit. Dogs were found infested by at least five species, i.e., *R. sanguineus* s.l., *R. turanicus* and three (i.e., *R*. sp. I in Italy (south) and Greece, *R*. sp. II in Italy (north), Spain and Portugal, and *R*. sp. III in India and Pakistan) of the four aforementioned OTUs (Table 1).

*Rhipicephalus sanguineus* s.l. was found in all continents, in at least 11 countries: Australia, Brazil, Colombia, Costa Rica, France, Guatemala, Honduras, India, South Africa, Thailand, and Vietnam.

## Discussion

The general morphological similarity among some *Rhipicephalus* species examined herein (e.g., *R. sanguineus* s.l., *R.* sp. I, and *R*. sp. II) was evident. However, a careful morphological examination of key characters (e.g., female genital opening shape, male adanal plate shape, scutal punctuation pattern and spiracular plate shape in both sexes) enabled us to separate these species. Importantly, measurements of adults are known to be of limited use for the differentiation of *Rhipicephalus* species [16]. However, considering the differences found herein for some particular characters (e.g., adanal plate length/breadth ratio) (Table 3), future studies should better assess the usefulness of morphometrical analysis for the differentiation of *Rhipicephalus* adults and immature stages.

Our genetic analyses further confirmed the differentiation of all the rhipicephaline species herein examined by supporting the existence of well-defined clades. A high genetic convergence was detected within and among populations of ticks identified as *R. sanguineus* s.l., irrespective of their geographical origin. This was also inferred based on intra- and interspecific nucleotide variations of mitochondrial DNA, which clearly defined the molecular identity of *R. sanguineus* s.l. (see Tables 4 and 5). In particular, the maximum nucleotide variation within each *Rhipicephalus* species (from different geographical areas) was 2.2%, 2.5%, and 3.5% for 16S rDNA, 12S rDNA and *cox*1, respectively. These percentages are lower than those reported in a previous study (i.e., up to 2.7% and 6.6% for 16S and 12S rDNA, respectively) using Brazilian ticks identified as *R. sanguineus*[19]. Furthermore, an overall intraspecific variation ranging from 7.8% to 8% was recorded for 12S rDNA sequences by other authors [17,18], which indicates that these authors were dealing with more than one species. In fact, the existence of different species under the name ‘*R. sanguineus*’ has recently been confirmed by studies carried out in South America, United States and China [18-22,27,48].

Recent assessments of the genetic variability of different *R. sanguineus* s.l. populations have provided new insights into the biosystematic status of this tick group and revealed the existence of at least two divergent populations [19-22,27]. Sequences of *R. sanguineus* s.l. herein obtained clustered within the so-called northern lineage whereas those of *R*. sp. II corresponded well to the southern lineage. Interestingly, the other three OTUs identified herein formed independent lineages, suggesting that they may represent separate species. As a corollary, crossbreeding studies have provided evidence that some of the taxa currently identified as *R. sanguineus* s.l. may actually represent separate species [12,18,21]. Moreover, a recent comparison between the complete mitochondrial genome of ‘*R. sanguineus*’ from China and USA revealed a nucleotide sequence difference in the whole mitochondrial genome of 11.23% between them [27]. This demonstrates that the two aforementioned populations represent two separate species. Nonetheless, without a type specimen and a consensual description, it is difficult to ascertain which one represents the true *R. sanguineus* s.s.

The widespread use of mitochondrial DNA in phylogenetic and population genetic studies results from a relatively high mutation rate and the apparent simplicity of mitochondrial maternal inheritance compared to the nuclear DNA [49]. However, paternal leakage, heteroplasmy and recombination have now been documented in multiple systems [49]. These exceptions to the key principles of mitochondrial inheritance may affect phylogenetic and population genetic analyses and should be taken into account while examining the results. Importantly, knowledge of mitochondrial inheritance in ticks is incipient and therefore coupling genetic and morphological data becomes pivotal for a better definition of species. Furthermore, different species may potentially mate in the field and the existence of hybrids among field-collected tick specimens cannot be ruled out, mainly in areas where closely related species occur together (sympatrically).

The usefulness of combining different methodologies towards defining integrated operational taxonomic units (IOTUs) has recently been exemplified elsewhere [50]. Our combined analyses provided evidence for the existence of at least four IOTUs among ticks examined in this study. Indeed, besides the morphological differences, the interspecific nucleotide variations for each genetic target analysed between *R. sanguineus* s.l. and these IOTUs were higher than between *R. sanguineus* s.l. and *R. guilhoni* (see Table 4).

Taxonomy is a fundamental part of science, without which human beings would not be able to recognize themselves as *Homo sapiens*. However, besides the taxonomic implications of data generated herein, our results will also raise new questions regarding the biology, ecology, vector competence and capacity of *R. sanguineus* s.l. to different pathogens worldwide [51-53]. For instance, it is widely believed that *R. sanguineus* s.l. is the vector of *Anaplasma platys*, but the only attempt to prove this hypothesis failed, which led to the conclusion that this tick may not act as a vector of this bacterium [54]. However, additional studies are needed to evaluate the vector competence of *R. sanguineus* s.l. and related species to transmit different pathogens (e.g., *A. platys*, *Babesia gibsoni*, *Ehrlichia canis*, and *Hepatozoon canis*) to dogs. These studies would also help decipher the relationship between *Rhipicephalus* spp. and the epidemiology of Mediterranean spotted fever [55].

Comparisons with the type specimens of *R. sanguineus* s.s. and *R. turanicus* were not possible. The types of *R. sanguineus* s.s. do not exist, whereas the male lectotype and the female paralectotype of *R. turanicus* are deposited in the tick collection of the Zoological Institute of the Russian Academy of Sciences, in St. Petersburg [56], being difficult to assess. Two of us (FD-T and DO) have recently visited the aforementioned collection and examined specimens labelled as ‘*R. turanicus*’ collected in Turkmenistan and determined by Dr. N. A. Filippova, a leading taxonomist that dedicated much of her life to the study of *Rhipicephalus* ticks. These specimens were morphologically and genetically compatible with our *R. turanicus* specimens from Italy and Greece, thereby allowing us to confirm the presence of this species in the Mediterranean region. Remarkably, our phylogenetic analyses pointed out the existence of different species under the name ‘*R. turanicus*’. For instance, 12S rDNA sequences from Zambia (DQ849231, DQ849232, DQ901290) and Zimbabwe (AF150017) labelled as ‘*R. turanicus*’ are closer to *R. sanguineus* s.l. than to *R. turanicus sensu* Filippova [14] (Figure 7). Similarly, some 12S rDNA sequences from Israel (AF150013), Kyrgyzstan (FJ536578), and Uzbekistan (FJ536579) labelled as ‘*R. turanicus*’ are most likely distinct species, as also inferred by pairwise comparisons of nucleotide sequences (data not shown). Finally, our ticks designated as *R*. sp. III (India and Pakistan) and *R*. sp. IV (Nigeria) were morphologically and genetically related to, but different from *R. turanicus*.

## Conclusions

The present study, along with recent morphological, biological, and genetic data produced by distinct research groups, irrefutably point out the existence of different species under the names ‘*R. sanguineus*’ and ‘*R. turanicus*’. Certainly, a re-description of *R. sanguineus* s.s. based on detailed morphological, biological and genetic data is fundamental. This will also allow us to re-examine ticks currently placed in synonym with *R. sanguineus* s.s. (e.g., *Rhipicephalus siculus* Koch 1844), some of which may need to be resurrected. Indeed, some of the four IOTUs described herein may actually represent species that have already been described and placed in synonym with *R. sanguineus* s.s. or, eventually, new species. Definitely, until the species is re-described based on a consensus among taxonomists, the use of the name *R. sanguineus* s.s. should be avoided.

## Competing interests

The authors declare there are no conflicts of interest.

## Authors’ contributions

FD-T and DO conceived the study. FD-T conducted the morphological study. MSL, GA and FD-T conducted the molecular study. AG and AP provided technical support and participated of field and/or laboratory activities. FD-T wrote the first draft of the manuscript and all authors approved the final version of the manuscript.
